# Relationship between vitamin B_12_, folate and homocysteine levels and *H. Pylori* infection in patients with functional dyspepsia: A cross-section study

**DOI:** 10.1186/1756-0500-5-206

**Published:** 2012-04-30

**Authors:** Shahid Rasool, Shahab Abid, Mohammad Perwaiz Iqbal, Naseema Mehboobali, Ghulam Haider, Wasim Jafri

**Affiliations:** 1 Section of Gastroenterology, Department of Medicine, Aga Khan University, Karachi, 74800, Pakistan; 2 Department of Biological & Biomedical Sciences, Aga Khan University, Karachi, 74800, Pakistan

## Abstract

**Background:**

*H. pylori* infection has been associated with many micronutrient deficiencies. There is a dearth of data from communities with nutritional deficiencies and high prevalence of *H. pylori* infection. The aim of this study was to determine the impact of *H. pylori* infection on serum levels of vitamin B_12_, folate and homocysteine in patients with functional dyspepsia (FD).

**Methods:**

One hundred and thirty-two patients with FD undergoing gastroscopy were enrolled. The serum was analyzed for B_12_, folate and homocysteine levels before gastroscopy. *H. pylori* infection was diagnosed by histopathological examination of gastric biopsies and urea breath test. An independent sample *t*-test and the Mann–Whitney test were used to compare mean serum concentrations of biomarkers between *H. pylori-*positive and *H. pylori-*negative groups of patients. A Chi-square test was performed to assess the differences among proportions, while Spearman’s rho was used for correlation analysis between levels of B_12_ and homocysteine.

**Results:**

The mean age of the group was 40.3 ± 11.5 (19–72) years. Folate deficiency was seen in 43 (34.6%), B_12_ deficiency in 30 (23.1%) and hyperhomocysteinemia in 60 (46.2%) patients. *H. pylori* was present in 80 (61.5%) patients with FD while it was absent in 50 (38.5%). Mean serum levels of B_12,_ folate and homocysteine in the *H. pylori-*positive group of patients were not significantly different from the levels in the *H. pylori-*negative group (357 ± 170 vs. 313 ± 136 pg/mL; p = 0.13), (4.35 ± 1.89 vs. 4.42 ± 1.93 ng/mL; p = 0.84); (15.88 ± 8.97 vs. 16.62 ± 7.82 μmol/L; p = 0.24); respectively.

B_12_ deficiency (≤200 pg/mL) was 23.8% in the *H. pylori-*positive patients versus 22.0% in the *H. pylori-*negative patients. Folate deficiency (≤3.5 ng/mL) was 33.8% in the *H. pylori-*positive group versus 36% in the *H. pylori-*negative group. Hyperhomocysteinemia (>15 μmol/L) was present in 46.2% of *H. pylori-*positive patients compared to 44% in the *H. pylori-*negative group. Correlation analysis indicated that serum B_12_ levels were inversely associated with serum levels of homocysteine in patients with FD (rho = −0.192; p = 0.028).

**Conclusions:**

This study demonstrated an inverse relationship between serum levels of B_12_ and homocysteine in patients with FD. Moreover, no impact of the presence of *H. pylori* was found on B_12_, folate and homocysteine levels in such patients.

## Background

*Helicobacter pylorus* is a gram negative, microaerophilic human pathogen which is prevalent worldwide. According to some community-based studies, more than half of the adult population in developed countries and 90% of those in developing countries harbor this bacterium [1,2].

Based on these reports, it is conceivable that the prevalence of *H. pylori* would be very high in the Pakistani population. Unfortunately, no community-based studies have been carried out in Pakistan. However, hospital-based data of dyspeptic patients indicate that the prevalence of *H. pylori* in Pakistan is about 80% [3].

*H. pylori* infection causes gastritis and it is associated with the development of peptic ulcer disease, gastric carcinoma and micronutrient deficiencies [4]. Micronutrient deficiencies may present with clinical syndromes ranging from subtle sub-clinical states, dysmotility like dyspepsia or severe clinical neurological and hematological disorders [5]. A recent review of a number of published studies on the influence of *H. pylori* on nutritional status revealed that the infection appeared to have a definite negative effect on vitamin B_12_ and vitamin C metabolism [6]. In a study from the Aga Khan University, high prevalence values of vitamin B_12_ and folate deficiencies, along with hyperhomocysteinemia (>15 μmol/L), were seen in Pakistani patients with acute myocardial infarction [7]. Hyperhomocysteinemia and high prevalence of folate deficiency were also observed in normal healthy subjects [7-9]. FD is a very common symptom in the community. Whether *H. pylori* infection has a role in folate and B_12_ deficiency in dyspeptic patients is still controversial.

### *H. Pylori* infection, folate and B_12_ deficiencies and hyperhomocysteinemia

*H. pylori* have been associated with coronary heart disease [10]. Hyperhomocysteinemia secondary to folate and B_12_ deficiency might be the link between *H. pylori* infection and coronary heart disease. Reduced folate and B_12_ absorption can occur in an environment of increased gastric juice pH. This would result in a reduced folate status leading to decreased activity of methionine synthase and increased serum concentration of homocysteine. Homocysteine is toxic to endothelial cells and a risk factor for atherosclerosis [11]. Since 1994, several studies have been published on B_12_ and folate levels in *H. pylori* infected patients with conflicting results [6,12,13]. A review based upon more than two dozen studies dealing with *H. pylori* infection and vitamin B_12_ status and *H. pylori* infection and hyperhomocysteinemia or both failed to show any clear relationship among *H. pylori* infection, B_12_ deficiency and hyperhomocysteinemia [14]. Hence the objective of this study was to investigate the relationship between folate, vitamin B_12_ and homocysteine levels and the impact of *H. pylori* infection on this relationship in patients with FD.

## Methods

### Study population

One hundred and thirty-two consecutive adult males and non-pregnant females with symptoms of dyspepsia who were undergoing gastroscopy were enrolled in the study at the Aga Khan University Hospital (AKUH) from Jan 2006 - Jan 2008. Prior written informed consent was obtained from all the study patients. FD was defined as the presence of one or more symptoms of epigastric pain, postprandial bloating, epigastric burning and/or early satiety that are considered to originate from the gastroduodenal region in the absence of any organic, systemic or metabolic disease. Before inclusion in the study, patients were screened for thyroid dysfunction (by determining the serum levels of triiodothyronine, thyroxine and thyroid stimulating hormone), gall bladder disease (by ultrasound), diabetes mellitus (by determining fasting serum levels of glucose) and hepatitis B and C (by determination of serum antibodies). Moreover, patients currently using proton pump inhibitors, strict vegetarians, alcoholics, and other comorbids like chronic liver disease, chronic renal failure, and mal-absorption syndrome were excluded from the study. Patients with a recent history of *H. pylori* eradication therapy (during a period of six months prior to the study) were also excluded. Pregnant females were excluded due to their physiological state and contraindication of the urea breath test. Patients with a history of folic acid and B_12_ supplementations during the six months prior to the study were also excluded. A total number of 14 patients were excluded on the basis of above mentioned criteria. In the study, we did not include healthy Pakistani adults (as a negative control group) because 3 studies on healthy adults in Karachi over the past few years provided us with sufficient evidence about the prevalence estimates of vitamin B_12_ deficiency, folate deficiency and hyperhomocysteinemia [7-9]. The study was approved by the Ethics Review Committee of the Aga Khan University.

### Determination of serum levels of vitamin B_12_, folate and homocysteine

Prior to the gastroscopy, a fasting venous sample of 5 mL of blood was taken to check serum B_12_ and folate and homocysteine levels. Serum samples were analyzed for folate and vitamin B_12_ using radio-assays [15,16].

Brief details of these procedures have been given below:

Vitamin B_12_ (cobalamin) assay:

Vitamin B_12_ (cobalamin) in serum sample was extracted by using one volume of serum, one volume of 0.06 M citrate-phosphate buffer, pH 2.6 and one volume of KCN (100 μg/mL) in a bath of boiling water for 30 min. After centrifugation, the clear supernatant solution was neutralized with 0.2 M Na_2_HPO_4_, pH 9.0.

In this competitive ligand binding assay, [^57^Co]cyanocobalamin [MP Biomedicals, LLC, USA] was used as the radiolabelled ligand and chicken serum as a binder of vitamin B_12_ in a dilution which approximately binds 50% of the radiolabelled cyanocobalamin. 2.5% hemoglobin-coated charcoal was used to separate unbound vitamin B_12_ (labelled as well as unlabelled) from the vitamin bound to the binder.

A reaction mixture for the standard curve contained 0.7 mL of 0.06 M citrate-phosphate buffer, pH 7.4, containing 70 μg KCN and Ringer’s bicarbonate (110 mM NaCl, 2 mM NaHCO_3_, pH 7.4); 0.1 mL [^57^Co]cyanocobalamin (15 pg); 0.1 mL cyanocobalamin (0‐40 pg) or the unknown sample extract and 0.1 mL of chicken serum diluted to bind approximately 50% of the radiolabelled cyanocobalamin. After 30 min incubation at room temperature in the dark, the reaction was stopped in each tube by adding 2.5% hemoglobin-coated charcoal and incubating it at 4°C for 5‐10 min with intermittent shaking followed by centrifugation at 3000 rpm for 15 min. Radioactivity in supernatant fraction (1 mL) was counted in a Gamma Counter (Genesys Gamma 1, Laboratory Technology Inc., Maple Park, IL, USA). A standard dose–response curve was generated by plotting % bound radioactivity versus pg amount of cyanocobalamin and the concentration of cobalamin in the unknown sample was determined by reference to this standard curve. The minimum limit of detection for cobalamin by this method is 50 pg/mL.

#### Folate (PteGlu) assay

Folate in the serum was assayed using a noncompetitive ligand binding radioassay. In this assay, radiolabelled pteroylglutamic acid ([^3^ H] PteGlu; Amersham Pharmacia, Buckinghamshire, UK) was used as the tracer and methyl-H_4_PteGlu (Sigma Chemical Co., St. Louis, MO, USA) as the unlabelled ligand and β-lactoglobulin (Sigma Chemical Co., St. Louis, Mo, USA) as the binder. A typical reaction contained 0.2 mL of 0.05 M borate-Ringer’s buffer, pH 8.0 with ascorbic acid (2 mg/mL); 0.1 mL of methyl-H_4_PteGlu (50‐300 pg) or 0.02 mL of serum sample and 0.1 mL of β-lactoglobulin (binder that had been diluted to bind 50‐60% of the tracer used in the assay) in a total reaction volume of 0.4 mL.

The reaction mixture was incubated at room temperature for 30 min and then the tubes were cooled to 4°C by placing them in an ice bath for 30 min. After this incubation, 0.1 mL of [^3^ H] PteGlu (200 pg) was added to each reaction tube and the incubation at 4°C was carried out for another 30 min. Then the reactions were stopped by adding 0.4 mL of cold 2.5% hemoglobin-coated charcoal. After thorough mixing, each reaction tube was subjected to centrifugation at 3000 rpm for 10 min at 4°C to pellet the charcoal. Radioactivity in the supernatant solution (0.5 mL) was counted in LS-6500 Spectrometer (Beckman Instruments, Palo Alto, CA, USA) using 5 mL of 3a70 scintillation fluor (Research Product International, USA). The standard dose–response curve was constructed by plotting the ratio of % [^3^ H] PteGlu bound (B) to % [^3^ H] PteGlu free (F) as a function of the amount of methyl-H_4_PteGlu in each standard. A blank consisting of 0.4 mL of 0.05 M borate-Ringer’s buffer, pH 8.0 with ascorbic acid and 0.1 mL of [^3^ H] PteGlu (200 pg) was also run to find out the radioactivity other than [^3^ H] PteGlu in the tracer. These counts were subtracted from each reaction. Concentration of methyl-H_4_PteGlu in the test sample was determined by reference to this standard dose–response curve. The minimum limit of detection for methyl-H_4_PteGlu by this method is 0.5 ng/mL.

Both the assay methods were validated by “recovery studies”. A known amount of cyanocobalamin or methyl-H_4_PteGlu was added to the human serum sample. Using the assay for cobalamin or folate, recovery of added vitamin was determined in the sample. For quality assurance in every assay, standard control serum samples obtained from Aga Khan University Hospital Clinical Laboratory containing the vitamin in low and high concentrations (relative to the normal range of levels of that vitamin) were run along with the standard curve. If the inter-assay variation in concentration of these controls was greater than 15%, the samples along with the standard curve were repeated.

Serum samples were screened for homocysteine using a kit method based on fluorescence polarization immunoassay (Abbott Laboratories, Ltd., Pakistan) following the manufacturer’s instructions. The minimum limit of detection for homocysteine by this assay is 4 μmol/L.

### Assessment of *H. Pylori* infection

In this study, *H. pylori* infection was defined by the demonstration of this bacterium on biopsies by two staining methods and positive ^14^ C] urea breath test [17].

Two biopsy samples were taken from the antrum and body of the stomach using a standard biopsy forceps for histopathological examination. These biopsy specimens were stained with Hematoxylin & Eosin and Giemsa stain for the detection of *H. pylori.* This is an established method for the detection of *H. pylori*[17]. After gastroscopy, ^14^ C] urea breath test (UBT) was performed for the rapid diagnosis of *H. pylori* infection. Microdose ^14^ C] UBT is claimed to be a reliable and rapid diagnostic test for *H. pylori* infection and it has been validated by several studies [18]. Patients positive with both methods were regarded as true positive, while those negative with both methods were regarded as true negative. If only one result was positive, that patient was excluded from the study.

The serum levels were dichotomized to separate variables to reflect the status, as vitamin B_12_ deficiency (≤200 pg/mL), folate deficiency (≤3.5 ng/mL) and hyperhomocysteinemia (>15 μmol/L). Patients were divided into two groups on the basis of the presence of *H. pylori* infection.

### Statistical analysis

The results are presented as means ± standard deviation, median with interquartile range (IQR) for most of the continuous variables and number (percentages) for categorical variables. Univariate analysis was performed by using a chi-square test to assess the difference among the proportions. An independent sample *t*-test was used to compare the difference of means if the independent variables followed normal distribution, otherwise the Mann–Whitney *U*-test (non-parametric) was performed for *H. pylori* positive and negative groups. Spearman’s rho was used for correlation analysis between vitamin B_12_ levels or folate levels and homocysteine concentration. A p-value < 0.05 was considered as statistically significant. All p-values were two sided. The Statistical Package for Social Science SPSS (Release 11.5.0, standard version, copyright© SPSS; 1989‐2002) was used for data analysis.

## Results

A total of one hundred and thirty two patients (85 males and 47 females) fulfilling the inclusion criteria were enrolled in the study. The mean age of the study group was 40.3 ± 11.5 years (range 19–72 years). *H. pylori* was present in 81 (61.4%) patients, while it was absent in 51 (38.6%). There were no significant differences in age and gender of *H. pylori* positive and negative patients. Complete biochemical data were available for 130 patients. Analysis of these data revealed that serum levels of vitamin B_12_, folate and homocysteine were not significantly different between *H. pylori* positive and *H. pylori* negative patients (Table 1). The mean serum concentration of total homocysteine in male patients was significantly higher than that of female patients (17.3 ± 9 vs. 14 ± 7.2 μmol/L; p = 0.02). However, in *H. pylori* positive patients a statistically significant difference was observed in homocysteine concentrations between males and females (p = 0.008; Table 2). Correlation analysis of total homocysteine concentration vs. age indicated no significant relationship (p = 0.77).

**Table 1:** **Vitamin B**_**12**_**, folate and homocysteine levels in *H. pylori *-positive and negative patients with functional dyspepsia**

**Variable**	**Concentration**		P value*
	***H. pylori *positive**	***H. pylori *negative**	
	**(n = 80)**	**(n = 50)**	
	**Mean ± SD/median (IQR)**	**Mean ± SD/median (IQR)**	
Vitamin B_12_ (pg/mL)	357 ± 170/350 (240)	313 ± 136/290 (170)	0.13
Folate (ng/mL)	4.35 ± 1.89/4.6 (2.85)	4.42 ± 1.93/4.5 (3.32)	0.84
Homocysteine (μmol/L)	15.88 ± 8.97/13.84 (8.97)	16.62 ± 7.82/14.5 (7.62)	0.24

*The Mann–Whitney test was used to compare mean homocysteine levels in *H. pylori* positive vs. *H. pylori* negative groups, while an independent sample *t* test was used for comparison of mean concentrations of vitamin B_12_ and folate between *H. pylori* positive and *H. pylori* negative groups.

**Table 2:** **Gender difference in homocysteine levels in patients with functional dyspepsia with and without *H. pylori *infection**

**Patient group**	**Homocysteine concentration (μmol/L)**				P value*
	**n**	**Males**	**n**	**Females**	
		**Mean ± SD/median (IQR)**		**Mean ± SD/median (IQR)**	
*H. pylori* positive (n = 80)	52	17.1 ± 9.43/15.4 (9.0)	28	13.58 ± 7.7/11.7 (7.94)	0.008
*H. pylori* negative (n = 50)	32	17.7 ± 8.4/15.2 (7.0)	18	14.8 ± 6.4/12.8 (9.42)	0.09

*The Mann–Whitney test was used to compare homocysteine levels in males and females in each group of patients.

Percent deficiencies of B_12_ and folate in patients with FD were found to be 23.1% and 34.6%, respectively. However, no difference was found between the proportions of the deficiencies of these two vitamins in *H. pylori* positive and *H. pylori* negative patients (p > 0.05; Table 3).

**Table 3:** **Hyperhomocysteinemia, B**_**12**_**and folate deficiencies in patients with functional dyspepsia with or without *H.pylori *infection**

**Parameter**	***H. pylori *positive**	***H. pylori *negative**
	**(n = 80)**	**(n = 50)**
	**n (%)**	**n (%)**
Vitamin B_12_ deficiency	19 (23.8)	11 (22)
(≤200 pg/mL)		
Folate deficiency	27 (33.8)	18 (36)
(≤3.5 ng/mL)		
Hyperhomocysteinemia	37 (46.2)	22 (44)
(>15 μmol/L)		

Percentages in *H. pylori* positive and *H. pylori* negative patients were compared by employing a test of association using chi-square.

Hyperhomocysteinemia was present in 46.2% of all patients. However, in patients with *H. pylori* infection, hyperhomocysteinemia was found in 37 (46.2%) of them as compared to 22 (44%) in the *H. pylori* negative group (p = 0.82). Correlation analysis (Spearman’s rho) indicated that serum B_12_ levels were inversely correlated with serum homocysteine in these patients with FD (rho = −0.192; p = 0.028). No statistically significant relationship between folate and/or B_12_ deficiency and serum levels of homocysteine was observed between the *H. pylori* positive and *H. pylori* negative patients (Table 4).

**Table 4:** **Relationships between B**_**12**_**, folate and homocysteine levels with *H. pylori *status in functional dyspepsia patients**

**Vitamin status**	***H. pylori *positive (n = 80)**			***H. pylori *negative (n = 50)**		P value*
	**n**	**Homocysteine (μmol/L)**	P value*	**n**	**Homocysteine (μmol/L)**	
		**Mean ± SD/median (IQR)**			**Mean ± SD/median (IQR)**	
Vit B_12_ deficient (≤200 pg/mL)	19	15.8 ± 7.3/15.5 (9.2)	0.68	11	18.1 ± 7.9/16.2 (7.0)	0.33
Vit B_12_ normal (>200 pg/mL)	61	15.9 ± 9.5/13.8 (8.2)		39	16.2 ± 7.9/14.2 (9.1)	
Folate deficient (≤3.5 ng/mL)	27	17.4 ± 7.7/15.3 (11.9)	0.07	18	19.6 ± 10.5/17.2 (8.9)	0.16
Folate normal (>3.5 ng/mL)	53	15.1 ± 9.5/13.1 (7.6)		32	14.9 ± 5.3/13.9 (5.8)	

*The Mann–Whitney test was used to compare mean homocysteine levels in vitamin deficient vs. vitamin normal groups.

## Discussion

B_12_ deficiency had been reported to be 6.6% and 9.7% in the normal healthy adult population of Pakistan, which is mostly non-vegetarian [7-9]. Compared to this, there was a high proportion of B_12_ deficiency (23.1%) in our patients with FD. Other studies from Pakistan and neighboring countries have also shown a high prevalence of B_12_ deficiency in the apparently healthy population [19,20]. Only two studies in Asia have been conducted which demonstrate B_12_ deficiency in patients with upper gastrointestinal symptoms [21,22]. These two studies attributed B_12_ deficiency to *H. pylori* infection. However, our results did not show similar involvement in Pakistani patients with dyspepsia.

Very high levels of homocysteine have been previously reported in healthy adult populations in Pakistan and neighboring India [8,9,19,23,24] with nutritional deficiency and lead pollution being possibly the major determinants. In the present study, hyperhomocysteinemia was found in 46.2% of patients with FD. This is a higher proportion as compared to 32% in an apparently healthy population study conducted in Karachi, Pakistan [8].

The role of *H. pylori* infection in micronutrient deficiencies is still not clear. The association of *H. pylori* infection and food cobalamin malabsorption has suggested to many investigators that *H. pylori* infection may predispose or even be causative in B_12_ deficiency [21,25,26]. *H. pylori* can cause B_12_ malabsorption by hypochlorhydria associated with atrophic gastritis which may lead to a failure in the splitting of B_12_ from food binders and its subsequent transfer to salivary R-binder in the stomach [6]. Another study demonstrated a decreased secretion of ascorbic acid from gastric mucosa and inactivation of intrinsic factor, and possibly leading to a decrease in B_12_ and folate absorption in patients who are harboring *H. pylori* infection. The ensuing deficiency of B_12_ and folate in such patients would lead to hyperhomocysteinemia [6,27].

Conflicting studies exist in the literature related to the effect of *H.pylori* eradication on serum B_12_ levels. Some of these have shown an improvement in vitamin B_12_ level after successful eradication of *H. pylori* infection or a positive association [13,28,29], while other found no relation between *H. pylori* infection and B_12_ deficiency [12]. Similarly, conflicting results have been reported regarding the effect of *H. pylori* on serum folate and homocysteine levels. Serum levels of folate were found to be significantly low and homocysteine levels significantly high in an *H. pylori* infected group of patients undergoing coronary arteriography [13]. In the present study, no differences were found in the concentrations of serum folate as well as homocysteine in *H. pylori* positive and *H. pylori* negative patients with FD. Our observations are supported by other studies demonstrating no clear association between *H. pylori* infection and serum folate and homocysteine levels [30-32].

## Conclusion

The present study demonstrated an inverse relationship between serum levels of vitamin B_12_ and homocysteine in patients with FD. Moreover, no impact of the presence of *H. pylori* was found on B_12_, folate and homocysteine levels in such patients.

## Competing interests

The authors declare that they have no competing interests.

## Authors’ contributions

SR, MPI, SA, WJ conceived and designed the study, interpreted the data and drafted the manuscript. NM and GH performed the experiments and analyzed the data. All authors read and approved the final version of the manuscript.

## Authors' information

SR: MBBS, FCPS; consultant Physician and Gastroenterologist, Aga Khan University, Karachi

SA: MBBS, FCPS, FRCP, PhD, Professor of Gastroenterology, Aga Khan University, Karachi

MPI: M.Sc, MS, PhD, Professor of Biochemistry, Aga Khan University, Karachi

NM: M.Sc, Research Coordinator, Aga Khan University, Karachi

GH: B.Sc, Research Associate, Aga Khan University, Karachi

WJ: MBBS, FRCP, FACP, FACG, Professor of Gastroenterology, Aga Khan University, Karachi
